# Exposure to tobacco smoke based on urinary cotinine levels among Israeli smoking and nonsmoking adults: a cross-sectional analysis of the first Israeli human biomonitoring study

**DOI:** 10.1186/1471-2458-13-1241

**Published:** 2013-12-30

**Authors:** Hagai Levine, Tamar Berman, Rebecca Goldsmith, Thomas Göen, Judith Spungen, Lena Novack, Yona Amitai, Tamy Shohat, Itamar Grotto

**Affiliations:** 1 Braun School of Public Health and Community Medicine, Hebrew University-Hadassah Faculty of Medicine, Kiryat Hadassah, Ein Kerem, Jerusalem, Israel; 2 The Hebrew University Center of Excellence in Agriculture and Environmental Health, Jerusalem, Israel; 3 Public Health Services, Ministry of Health, Jerusalem, Israel; 4 Institute and Outpatient Clinic of Occupational, Social and Environmental Medicine, University Erlangen-Nuremberg, Erlangen, Germany; 5 Faculty of Health Sciences, Ben-Gurion University of the Negev, Beer-Sheva, Israel; 6 Department of Management, Bar Ilan University, Ramat Gan, Israel; 7 Israel Center for Disease Control, Ministry of Health, Tel Hashomer, Israel; 8 Department of Epidemiology and Preventive Medicine, Tel Aviv University, Tel Aviv, Israel

**Keywords:** Epidemiology, Public health, Secondhand smoke, Exposure, Environmental tobacco smoke, Human Biomonitoring, Urinary cotinine

## Abstract

**Background:**

Cotinine levels provide a valid measure of exposure to environmental tobacco smoke (ETS). The goal of this study was to examine exposure to tobacco smoke among smoking and nonsmoking Israeli adults and to identify differences in ETS exposure among nonsmokers by socio-demographic factors.

**Methods:**

We analyzed urinary cotinine data from the first Israeli human biomonitoring study conducted in 2011. In-person questionnaires included data on socio-demographic and active smoking status. Cotinine levels were measured using a gas chromatography–mass spectrometry procedure. We calculated creatinine-adjusted urinary cotinine geometric means (GM) among smokers and nonsmokers, and by socio-demographic, smoking habits and dietary factors. We analyzed associations, in a univariable and multivariable analysis, between socio-demographic variables and proportions of urinary cotinine ≥1 μg/l (Limit of Quantification = LOQ) or ≥4 μg/l.

**Results:**

Cotinine levels were significantly higher among 91 smokers (GM = 89.7 μg/g creatinine; 95% confidence interval [CI]: 47.4-169.6) than among 148 nonsmokers (GM = 1.3; 1.1-1.7). Among exclusive waterpipe smokers, cotinine levels were relatively high (GM = 53.4; 95% CI 12.3-232.7). ETS exposure was widespread as 62.2% of nonsmokers had levels ≥ LOQ, and was higher in males (75.8%) than in females (52.3%). In a multivariable model, urinary cotinine ≥ LOQ was higher in males (Prevalence ratio [PR] = 1.30; 95% CI: 1.02-1.64, p = 0.032) and in those with lower educational status (PR = 1.58; 1.04-2.38, p = 0.031) and decreased with age (PR = 0.99; 0.98-1.00, p = 0.020, per one additional year). There were no significant differences by ethnicity, residence type or country of birth.

**Conclusions:**

Our findings indicate widespread ETS exposure in the nonsmoking Israeli adult population, especially among males, and younger and less educated participants. These findings demonstrate the importance of human biomonitoring, were instrumental in expanding smoke-free legislation implemented in Israel on July 2012 and will serve as a baseline to measure the impact of the new legislation.

## Background

Environmental tobacco smoke (ETS) is a combination of smoke emitted from a burning tobacco product and the smoke exhaled by the smoker, which is also called secondhand smoke [1].

The adverse effects of ETS among nonsmoking adults are well proven and mirror those associated with active smoking [2]. ETS is causally associated with lung cancer among never-smokers and among nonsmokers [3]. ETS increases the risk of cardiovascular disease by approximately 30% [4], and also increases the risk of respiratory diseases [5].

Various methods are available to measure ETS: self-reporting, environmental measurements and human biomonitoring of various biomarkers in different biological media [6]. Nicotine is a specific biomarker of exposure to tobacco smoke, either active or ETS, but due to its short half life (1-3 hours) it has limited value as a marker of exposure. Cotinine, the primary proximate metabolite of nicotine, is used most frequently as a biomarker of tobacco smoke exposure, as its half-life is longer (approximately 16-18 hours) and levels remain fairly constant during the day. The cotinine level provides a valid and quantitative measure of average recent human ETS exposure and is therefore the preferred biomarker of exposure to tobacco smoke in active smokers and in nonsmokers exposed to ETS [7]. Dietary intake of nicotine from food like fruits and vegetables is possible but likely to be negligible [8]. Urinary cotinine, especially when corrected for creatinine concentration, is highly correlated with plasma cotinine [9].

In Israel, according to the recent national survey, the overall active smoking rate in the adult (21 years and older) population, based on self-report, is 20.6% [10]. Smoking rates vary by sex and ethnicity: being highest among Arab males (43.8%) and lower for Jewish males (23.7%), Jewish females (15.9%) and Arab females (6.7%). There was a recent study on ETS exposure in public places in Israel based on air quality measurements [11]. However, assessment of ETS exposure of the Israeli population by human biomonitoring has never been conducted, thus the extent of exposure to ETS is unknown, both for the entire population and for specific sub-groups.

The goal of this study was to examine exposure to tobacco smoke among smoking and nonsmoking Israeli adults and to identify differences in ETS exposure among nonsmokers by socio-demographic factors, in order to plan tobacco control activities and to serve as a baseline for future monitoring.

## Methods

### Study design, settings and participants

The current study is based on the Israeli Human Biomonitoring Study which was a cross-sectional study on exposure of Israeli adults from the general population to environmental chemicals and/or their metabolites, as measured in urine samples. The primary objective of the biomonitoring study was to provide information on exposure to environmental chemicals in Israel in order to support public health policy. Aims and methods of the biomonitoring study are further detailed in our previous publications [12,13].

The eligible population included Israeli adults, aged 20-74, aiming to represent the Israeli non-institutionalized adult population. Recruitment, interviewing and sampling took place between February and June 2011. The potential sample size was 300 individuals, assuming up to 20% of missing data, incomplete questionnaires and invalid urine samples, to reach a planned sample size of 250 individuals. The parameters for defining the sample were selected so as to represent the population distribution of urban versus rural dwelling (with urban defined as population more than 2,000) and the two major ethnic groups in Israel (Jews and Arabs) as well as wide geographical representation. Overall, 20 cities/towns were selected, with 4 within the Arab sector (3 urban, 1 rural) and 16 within the Jewish sector (15 urban, 1 rural), representing the relative proportion of the ethnic groups in Israel. In each city/town, interviewers were requested to interview 15 people. Within each city/town, interviewers were required to select 5 separate areas. Within each area, recruitment was done by “knocking on doors” and interviewing those who met the inclusion criteria and agreed to participate, including providing a urine sample. The inclusion criteria were age (20–74) and ability to answer the questionnaire in Hebrew or Arabic. The response rate was 29%, excluding individuals not eligible for the study and individuals not at home at the time of the visit. People refusing participation were replaced by “knocking on the next door”. Participants were not targeted for specific health status and were not included or excluded on the basis of their potential for low or high exposures to environmental chemicals including tobacco exposure. Of 249 participants eventually included in the biomonitoring study, one was excluded from the present report due to a missing cotinine value.

The study was conducted in accordance with the ethical principles of the Declaration of Helsinki. The study protocol was reviewed and approved by the Sheba Tel Hashomer Helsinki Committee. Written informed consent was obtained for all respondents. Participation in the study was voluntary. At the time of recruitment participants received a note explaining that they would receive individual results on urinary concentrations of environmental contaminants if they requested it during their interview or if their individual urinary metabolite results were unusual (more than 10 times the 90^th^ percentile for the study population). All individuals receiving results were invited to contact the study coordinator for additional information. All analysis of data for the study was conducted without details on the identity of the participants.

### Data sources and variables

Study participants were interviewed using a structured questionnaire. The interviews were administered by trained interviewers. The interview consisted of a health and lifestyle questionnaire, including smoking habits, demographic questionnaire, a 24 hour dietary recall and a food frequency questionnaire. All completed questionnaires were returned to the Israel Center for Disease Control for data entry, quality assurance and analysis. Socio-demographic and personal variables included age (analyzed as a continuous variable, and also grouped as “younger”, 20-44 years, and “older” 45-74 years), sex (males/females), urbanicity (urban/rural), country of birth (Israel/other), education (lower education [high school level qualification or below]/higher education), and ethnicity (Jewish/Arab). Druze were grouped with Arab ethnicity. Twelve individuals had missing data for country of birth and four individuals had ethnicity other than Jewish or Arab, therefore they were excluded from the univariable analysis.

Smoking status was based on self-report. Questions used for active tobacco smoking status were: “Do you currently smoke, including hookah (water pipe)?”, “What do you currently smoke, or what did you smoke before? (cigarettes, cigars, pipe, waterpipe, other)”, “How many cigarettes do you smoke per day or per week?” Based on the first question, participants were classified as tobacco smokers (of any kind) or nonsmokers. Active smoking variables were smoking type (cigarettes or cigarettes and other forms/exclusive waterpipe/cigars or pipe) and cigarette smoking frequency (<10 cigarettes/day, 10-20 cigarettes/day, >20 cigarettes/day).

Urine spot samples were collected in 120-ml urine specimen containers. All urine samples were maintained at below 4°C for a maximum of 24 hours until they were transported to the Sheba Medical Center at Tel Hashomer. Urine samples were aliquoted at Sheba Medical Center and frozen at -20°C. Within four months of collection, urine samples were shipped to the University of Erlangen–Nuremberg in Germany on dry ice (-70°C), where they were analyzed.

Laboratory analyses of cotinine and creatinine were performed at the Institute and Outpatient Clinic of Occupational, Social and Environmental Medicine, University Erlangen-Nuremberg in Germany. Cotinine in urine was determined using a gas chromatography mass spectrometry procedure validated and published by the working group “Analyses in biological materials” [14]. In brief, cotinine was extracted from the urine using dichloromethane and quantified after gas chromatographic separation by mass spectrometry in single ion monitoring mode [15]. Deuterated cotinine was used as an internal standard. Calibration was performed using calibration standards which were prepared in pooled non-smoker urine and which were treated in the same manner as the samples to be analyzed. Limit of detection is 0.5 μg/liter and limit of quantification (LOQ) is 1 μg/liter. Limit of detection and limit of quantification were calculated based on a signal-to-noise ratio of 1 to 3 and 1 to 6, respectively. Urinary analyte concentrations were provided in units of μg/liter. These concentrations were divided by urinary creatinine concentrations (g creatinine/l urine) to generate creatinine-adjusted analyte concentrations. Creatinine in urine was determined by photometric detection as picrate according to the Jaffé method [16]. Quality control was performed by analysing aliquots of control material in each series and accuracy was validated by the successful participation in G-EQUAS for both parameters [17]. Concentrations below the LOQ for an analyte were replaced by the limit of detection (LOD). The main outcome variable was proportion ≥ LOQ. Secondary outcome was geometric mean (GM) of cotinine concentration adjusted for creatinine.

### Statistical methods

Characteristics of the study population and by smoking status were described by proportion for categorical variables and mean and standard deviation (SD) for continuous variables. Characteristics were compared between smokers and nonsmokers by Chi-Square or Fisher’s Exact test for categorical variables, and by t-test for continuous variables. GM and 95% confidence intervals (CI) were calculated for urinary creatinine-adjusted cotinine concentrations and compared between smokers and nonsmokers and between sub-groups of smokers and nonsmokers using the t-test procedure for ratio using the lognormal distribution.

Proportions of participants with cotinine levels ≥1 μg/l (LOQ) or among nonsmokers were described overall and by socio-demographic characteristics. We used the Chi-Square or Fisher’s Exact Test to compare the proportion of cotinine levels ≥ LOQ by socio-demographic characteristics. Associations between continuous variables such as age to creatinine-adjusted urinary cotinine among nonsmokers were assessed by Spearman correlation. Multivariable analysis was conducted using log-binomial regression with ≥ LOQ proportion as the outcome of interest and all significant (at p < 0.05) variables were introduced to the model. Similar univariable and multivariable analyses were conducted by a cut-off of ≥4 μg/l, representing higher ETS exposure, allowing comparison to a previous study in the German population [18], as well as descriptive analysis by different cut-off of 1.1 μg/l, allowing comparison with the Canadian Human Biomonitoring Study [19]. Prevalence ratio (PR) was used as a measure of association in all the analyses with p < 0.05 considered significant. To allow for comparison with other studies we calculated the odds ratio (OR) by logistic regression model as well, though the OR is expected to overestimate an effect due to the high prevalence of the outcome in the study (≥ LOQ proportion). All analyses were performed with SAS 9.2 (SAS Institute Inc., Cary, NC, USA).

## Results

The 248 study participants were divided into nonsmokers (n = 148) and smokers (n = 91) as 4 participants were excluded due to unverified smoking status and 5 additional participants were excluded due to discrepancy between self-reported smoking status as “nonsmokers” and creatinine-adjusted levels of cotinine >150 μg/g [20]. The average age of 148 nonsmokers (39.8 years, SD = 13.3) was significantly (p = 0.015) higher than that of the 91 smokers (36.0 years, SD = 10.8). Nonsmokers included a lower proportion of males (41.9% vs. 63.7%), Jewish ethnicity (69.7% vs. 83.3%) and rural residence (7.4% vs. 17.6%) compared to smokers (see Table 1).

**Table 1:** Characteristics of total study population and by smoking status, Israel human biomonitoring study, 2011

		**Total study population**^**a**^		**Non-smokers**^**b**^		**Smokers**		p-value^*^
		**(N = 248)**		**(N = 148)**		**(N = 91)**		
**Characteristic**		**N**	**% of total**	**N**	**% of total**	**N**	**% of total**	
**Age (years)**	20-44	164	66.1	90	60.8	68	74.7	0.027
	45-74	84	33.9	58	39.2	23	25.3	
**Sex**	Males	127	51.2	62	41.9	58	63.7	0.001
	Females	121	48.8	86	58.1	33	36.3	
**Ethnicity**	Jewish	184	74.2	101	69.7	75	83.3	0.019
	Arab	64	25.8	44	30.3	15	16.7	
**Urbanicity**	Urban	218	87.9	137	92.6	75	82.4	0.016
	Rural	30	12.1	11	7.4	16	17.6	
**Country of birth**	Israel	214	90.7	129	90.2	77	90.6	0.925
	Other	22	9.3	14	9.8	8	9.4	
**Education**	Lower education	189	76.2	111	77.1	72	79.1	0.714
	Higher education	55	22.5	33	22.9	19	20.9	

^*^For difference in characteristics between smokers and nonsmokers, significance determined using Chi-Square or Fisher’s Exact test.

^a^Data were missing for Country of Birth (12 participants) and Education (4 participants). 4 participants were not Jewish or Arabs and were excluded from ethnicity analysis.

^b^The smoking status could not be verified for 4 participants in the study -who were excluded from further analysis. 5 participants were excluded from the analysis due to discrepancy between self-reported smoking status as “non-smokers” and creatinine-adjusted levels of cotinine > 150 μg/g.

**Figure 1:**
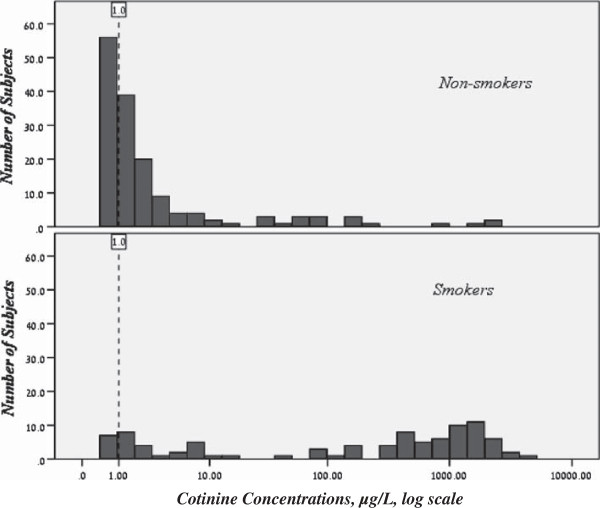
Distribution of urinary cotinine concentrations in Israeli smokers (n = 91) and non-smokers* (n = 153), Israel human biomonitoring study, 2011. Limit of quantification appears as dashed line. *Five nonsmoker participants with creatinine-adjusted cotinine concentrations >150 μg/g were excluded from further analysis.

All participants, smokers and nonsmokers, had urinary cotinine levels > LOD. The distribution of cotinine concentration levels (μg/liter) among smokers and nonsmokers reflects clear differences (see Figure 1). Urinary cotinine concentrations were significantly higher among smokers (GM = 89.7 μg/g creatinine; 47.4-169.6) than among nonsmokers (GM = 1.3; 1.1-1.7). Among the smokers, the majority (84.6%) smoked cigarettes (or cigarettes and other forms) and the minority were exclusive waterpipe users (14.3%) or cigar smokers and pipe users (1.1%). Cotinine levels were significantly higher among smokers who reported >20 cigarettes/day (GM = 328.2; 67.7-1591.7, PR = 5.86; 95% CI 1.11-30.85, p = 0.040) as compared to smokers who reported <10 cigarettes/day (GM = 53.0; 18.3-153.0), and not significantly higher as compared to those who reported 10–20 cigarettes/day (GM = 73.5; 22.3-242.9). Among the group of exclusive waterpipe smokers, all had levels above LOQ and cotinine levels were relatively high (GM = 53.4; 95% CI 12.3-232.7) and not significantly different than in cigarette smokers (GM = 89.3; 42.5-187.6).

Among the nonsmokers, 62.2% had levels ≥ LOQ and 16.2% had levels ≥4 μg/l (see Table 2). Proportions ≥ LOQ were significantly higher for males compared to females (75.8% vs. 52.3%, p = 0.003) and among subjects with lower education level compared to higher education level (69.4% vs. 42.4%, p = 0.021). In the younger age group (20-44 years), 67.8% had levels ≥ LOQ compared to 53.5% for the older age group (45-74 years, p = 0.096); there was a significant inverse association with age as a continuous variable (PR = 0.99; 95% CI 0.98-1.00, per one additional year, p = 0.022)., There was a tendency for a higher proportion of nonsmokers with cotinine levels above a cut-off point of ≥4 μg/l by education status (20.7% vs. 3.0%, p = 0.055) and by sex (22.6% vs. 11.6%, p = 0.08).

**Table 2:** Associations of socio-demographic factors with creatinine-adjusted cotinine concentrations in Israeli nonsmoking adults, Israel human biomonitoring study, 2011 (N = 148)

**Variable**		**≥1 μg/l (LOQ)**	**PR (95% CI)**	p-value*	**≥4 μg/l**	**PR (95% CI)**	p-value*	**Geometric mean (μg/g Creatinine)**	**95% CI**
		**% (n/N)**^**a,b**^			**% (n/N)**^**a**^				
**All**		62.2 (92/148)			16.2 (24/148)			1.33	1.06–1.67
**Age (years)**	20–44	67.8 (61/90)	Ref.		15.6 (14/90)	Ref.		1.27	0.95–1.68
	45–74	53.5 (31/58)	0.70 (0.60; 1.04)	0.096	17.2 (10/58)	1.11(0.53; 2.33)	0.786	1.42	0.96–2.12
**Sex**	Males	75.8 (47/62)	1.45 (1.13; 1.85)	0.003	22.6 (14/62)	1.94 (0.92; 4.08)	0.080	1.65	1.10–2.48
	Females	52.3 (45/86)	Ref.		11.6 (10/86)	Ref.		1.13	0.87–1.48
**Ethnicity**	Jewish	65.4 (66/101)	1.18 (0.88; 1.58)	0.266	17.8 (18/101)	1.40 (0.59; 3.29)	0.455	1.39	1.06–1.83
	Arab	52.3 (23/44)	Ref.		13.6 (6/44)	Ref.		1.17	0.73–1.86
**Residence type**	Urban	62.0 (85/137)	Ref.		15.3 (21/137)	Ref.		1.30	1.04–1.63
	Rural	63.6 (7/11)	1.03 (0.64; 1.63)	0.915	27.3 (3/11)	1.78 (0.63; 5.05)	0.279	1.69	0.38–7.52
**Country of birth**	Israel	59.7 (77/129)	Ref.		14.7 (19/129)	Ref.		1.23	0.97–1.56
	Other	71.4 (10/14)	1.20 (0.83; 1.72)	0.329	14.3 (2/14)	0.97 (0.25; 3.74)	0.965	1.36	0.77–2.41
**Education**	Lower education	69.4 (77/111)	1.64 (1.08; 2.48)	0.021	20.7 (23/111)	6.84 (0.96; 48.74)	0.055	1.58	1.20–2.07
	Higher education	42.4 (14/33)	Ref.		3.0 (1/33)	Ref.		0.79	0.53–1.19

*Significance levels are based on Univariable log-binomial regression, compared to reference level.

^a^For each variable category, the proportion is given as well as, in parentheses, the number of participants above the cut-off (n) and the total number of participants in the category (N).

^b^CI, Confidence interval; LOQ, Limit of quantification; PR, Prevalence ratio; Ref. = Reference.

There was no significant difference in the proportion ≥ LOQ or ≥4 μg/l or the GM by country of birth, ethnicity or urbanicity.

In a mutually adjusted model (see Table 3), urinary cotinine proportion ≥ LOQ remained associated with being male (PR = 1.30; 95% CI: 1.02-1.64, p = 0.032), inversely associated with age (PR = 0.99; 0.98-1.00, p = 0.020, per one additional year) and associated with having lower educational status (PR = 1.58; 1.04-2.38, p = 0.031). The inference based on the OR estimates in a mutually adjusted logistic regression model was similar to the finding of a log-binomial model, but as expected overestimated the magnitude of associations, i.e. male (OR = 2.56; 95% CI: 1.18-5.55), younger age (OR = 0.96; 0.94-0.99, per one additional year) and lower education (OR = 3.25; 1.39-7.59).

**Table 3:** **Associations of socio-demographic factors with urinary cotinine levels ≥ LOQ**^**a **^**(1 μg/l) in Israeli nonsmoking adults (n = 144), multi-variable log-binomial regression model, Israel human biomonitoring study, 2011**

**Parameter**	**p-value**	**PR (95% CI)**
**Males vs. Females**	0.032	1.30 (1.02; 1.64)
**Age, in years (per one additional year)**	0.020	0.99 (0.98; 1.00)
**Lower education vs. higher education**	0.031	1.58 (1.04; 2.38)

^a^CI, Confidence interval; LOQ, Limit of quantification; PR, Prevalence ratio.

As a sensitivity analysis, we further excluded 5 participants with self-reported smoking status as nonsmokers and creatinine-adjusted levels of cotinine >50 μg/g (but <150 μg/g). Findings were similar in the mutually adjusted model: associated with being male (PR = 1.28; 95% CI: 1.00-1.63, p = 0.048), inversely associated with age (PR = 0.99; 0.98-1.00, p = 0.014, per one additional year) and associated with having lower educational status (PR = 1.55; 1.03-2.35, p = 0.037).

## Discussion

The present study, the first to evaluate exposure to tobacco smoke using a biological marker in an Israeli general population, demonstrates widespread and high ETS exposure in the non-smoking adult population in Israel. Despite limited power due to small sample size, ETS exposure was found to be significantly more common among males, younger study participants and those with lower education.

We found clear differences in urinary cotinine levels between smokers and nonsmokers, with higher levels by smoking frequency. This expected finding gives some validation to our study methods such as data collection and laboratory analysis. Cotinine levels among exclusive waterpipe users were similar to those in cigarette smokers, in line with known limited data indicating that daily use of waterpipe produces nicotine absorption of a magnitude similar to that produced by daily cigarette use [21]. This observation gives further evidence to the addictive potential of this common habit in our population. There is a gradual increase in evidence related to the harmful effects of waterpipe smoking, as was suggested by our previous findings in Israel [22]. The effects of waterpipe passive smoking are largely unknown, although a recent Israeli study showed some physiological effects [23].

In our study all nonsmokers had levels of urinary cotinine above LOD, 62.2% had levels ≥1 μg/liter and 16.2% had levels ≥4 μg/liter. Our results are comparable to those in a previous study conducted in the German population in 1998, which found urinary cotinine levels ≥4 μg/liter in 23% of the nonsmokers and even higher in males (26%) as compared to females (19%) [18]. In a recent study in the Canadian general population 15% of those aged 20-39 years and 11% of those aged 40-59 years had urinary cotinine levels >1.1 μg/liter [19]. In our population, using the same cut-off, rates were at least four times higher in both age groups (67% and 45%, respectively). Median or geometric mean levels could not be compared as those were not reported in the Canadian or German studies due to a high proportion below the LOD, while in our study all nonsmokers had levels above LOD. In a finding similar to that in our study, widespread ETS exposure was previously found among nonsmokers in another Middle-Eastern country–Syria [24]. In that study, a moderate correlation was found between salivary cotinine levels and self-reported exposure measures such as house smoking policy. Our study gives further evidence to the widespread and high level of ETS exposure in the Middle East region.

The proportion of population exposed to ETS in our study, based on urinary cotinine concentrations ≥ LOQ, was much higher in males compared to females, a finding consistent with previous studies in the adult population [18,19,25,26]. Lower education and younger age were also important risk factors for exposure and remained so after mutual adjustment, consistent with studies in other countries [26-29].

In order to supplement our study, we analyzed data on self-reported ETS among nonsmokers, based on the 2010 Israel Central Bureau of Statistics Social Survey [30]. The survey was conducted by interviewing ~7500 Israelis during 2010, a random sample of the adult Israeli population. In this survey, 69.3% of nonsmokers aged 20-74 reported exposure to ETS, higher (74.3%) for males compared to females (65.5%). Among those who reported exposure, 33.6% reported ETS at the workplace, 26.8% at home, and 77.3% in other places. A much higher percentage of males reported ETS at work (46.7%) than females (22.3%). In contrast, a higher proportion of females reported ETS at home (35.4%) compared with males (16.8%). This analysis gives further evidence to the widespread ETS exposure in the Israeli population found in our study. Additional support for widespread ETS exposure stems from a study conducted among Israeli adolescents, based on self-reported exposure [31].

Public health and health promotion efforts regarding tobacco smoke exposure should be directed to the young male population, as not only are active smoking rates high, but ETS is also high and common among nonsmokers in this population. This group needs tailored interventions, to address social needs as well as attitudes to ETS which may include more tolerance to exposure at work.

The main finding of the study, widespread and high ETS in all groups of the Israeli population, has served to raise support and political will for taking further steps to reduce ETS in the public and private arenas in Israel. The present study was instrumental in expanding smoke-free legislation implemented in Israel in July 2012. This new legislation included prohibition of smoking, for the first time, in open spaces such as outside entrances to health facilities, railway platforms, etc. [32].

There are major problems in implementation of smoke-free legislation in bars and pubs in Israel due to social norms [33], despite the fact that the majority (67%) of Israelis support completely smoke-free bars and pubs, according to a previous survey [34]. The new popular trend of e-cigarettes (battery-operated products that deliver nicotine, flavor and other chemicals via a vapor that is inhaled by the user) in Israel and elsewhere should be taken into account when planning tobacco control policy, as it may reduce ETS but may give legitimacy to smoking in public places [35]. There is evidence that mobile health (m-Health) efforts for smoking cessation can be effective and mobile phones should be explored as a method for preventing ETS as well [36]. We are currently developing a text messaging program for Israeli smokers as well as a mobile phone application for reporting violations of the smoke-free law in public places in Israel.

National human biomonitoring studies on ETS exposure using cotinine measurements have been used by other countries to demonstrate the positive effects of smoke-free legislation and identify its impact on different population groups [25,26]. Cotinine biomonitoring in the general population is an important tool for tobacco control. Conducting national biomonitoring programs can be used to determine the average (and range of) ETS, establish baseline levels of exposure and assess trends over time, provide information for directing public health priorities for environmental health policy, including ETS, support and direct future research and identify potentially vulnerable groups. Urinary cotinine monitoring can also be used to evaluate the success of public health interventions. Such programs exist in the United States, Canada, Belgium, France, Germany and the European Union. Active tobacco smoking and ETS are important exposures in themselves, as well as confounders/mediators in many other environmental exposures, and should be assessed in depth in any national human biomonitoring program. We plan to conduct a follow up biomonitoring study in Israel in 2015 on exposure to ETS (as well as pesticides) in Israel, in order to determine whether the policies described above have been effective in reducing public exposure to ETS in the general population.

Two major strengths of the current study include the representation of a wide geographical distribution and different socio-demographic groups and the use of a valid, sensitive and accurate quantitative biochemical assessment of ETS. Cotinine was shown in the past to be derived from exposure to tobacco smoking, while other sources, such as dietary sources, were shown to be negligible [8]. Indeed, we did not find an association between urinary cotinine and fruits or vegetables consumption in our population (data not shown).

Nonetheless, some limitations of the study should be considered. An important methodological limitation of the study is the “convenient” non-random sampling technique. It is not certain to what extent the study population is representative of the general adult population in Israel. For example, the Arab population was over-represented in the study (25%) compared to 18% in the Israeli population of the same age [37]. Hence, interpretation of the findings should be done cautiously, although selection bias is an unlikely explanation for the widespread high ETS exposure found. A second limitation of our study is the lack of self-reported ETS status and sources, which could have helped in elaboration of ETS patterns. However, urinary cotinine levels give better estimates of ETS and we were able to confirm our findings by data from the 2010 Social Survey. Another limitation is the possibility of that cotinine levels found in our nonsmoking population derived not only from ETS, but, in some cases, from misclassification of smoking status or use of nicotine replacement therapy as a source of exposure. We minimized this possibility by exclusion of those with cotinine levels >150 μg/g and by our sensitivity analysis further excluding cotinine levels >50 μg/g. Unfortunately, we did not collect data on time elapsed since last cigarette smoked among self-reported smokers which might explain why some self-reported smokers (who did not smoke recently) had relatively low urinary cotinine levels as well as the higher smoking rate in our study population compared to the general Israeli adult population. Self-reported smoking status is considered valid, especially for those aged 20 and above [38], so it is unlikely that misclassification by smoking status significantly affected our results. Finally, our relatively small sample size gives limited power to identify differences in magnitude of exposure as expressed by GM, or between specific sub-groups such as those based on ethnicity.

## Conclusions

This study shows widespread ETS exposure in the nonsmoking adult Israeli population, especially among males, the younger population and those with lower education. These findings demonstrate the importance of human biomonitoring, were instrumental in expanding smoke-free legislation implemented in Israel on July 2012 and will serve as a baseline to measure the impact of the new legislation.

## Abbreviations

CI: Confidence interval; ETS: Environmental tobacco smoke; GM: Geometric mean; LOD: Limit of detection; LOQ: Limit of quantification; OR: Odds ratio; PR: Prevalence ratio; SD: Standard deviation.

## Competing interests

The authors declare that they have no competing interests.

## Authors’ contributions

HL led the analysis and interpretation, and drafted the manuscript. He takes responsibility for the integrity of the data and the accuracy of the data analysis. TB, RG and IG conceived the study, and participated in its design and coordination. YA and TS contributed to conception, design and acquisition of data. TG contributed to the design and led the laboratory analysis. JS and LN performed the statistical analysis. All authors read, revised and approved the final manuscript.

## Pre-publication history

The pre-publication history for this paper can be accessed here:

http://www.biomedcentral.com/1471-2458/13/1241/prepub
